# Isolation and characterization of low pathogenic H7N7 avian influenza virus from a red-crowned crane in a zoo in South Korea

**DOI:** 10.1186/s12917-020-02645-4

**Published:** 2020-11-10

**Authors:** Young-Jae Si, Yu-Na Lee, Sun-Ha Cheon, Yu-Ri Park, Yoon-Gi Baek, Soo-Jeong Kye, Myoung-Heon Lee, Youn-Jeong Lee

**Affiliations:** Avian Influenza Research & Diagnostic Division, Animal and Plant Quarantine Agency, 177, Hyeoksin 8-ro, Gimcheon-si, Gyeongsangbuk-do 39660 Republic of Korea

**Keywords:** AI surveillance, Biosecurity measure, Genetic analysis, H7N7, LPAI in a zoo

## Abstract

**Background:**

South Korea conducts annual national surveillance programs to detect avian influenza (AI) in domestic poultry, live bird markets, and wild birds. In March 2017, an AIV was isolated from fecal samples in an outdoor aviary flight cage in a zoo in Korea.

**Results:**

Nucleotide sequencing identified the isolate as low pathogenic avian influenza virus (LPAIV) H7N7, and DNA barcoding analysis identified the host species as red-crowned crane. This isolate was designated A/red-crowned crane/Korea/H1026/2017 (H7N7). Genetic analysis and gene constellation analysis revealed that A/red-crowned crane/Korea/H1026/2017 (H7N7) showed high similarity with four H7N7 LPAIVs isolated from wild bird habitats in Seoul and Gyeonggi in early 2017.

**Conclusions:**

Considering the genetic similarity and similar collection dates of the viruses, and the fact that zoo bird cages are vulnerable to AIV, it is likely that fecal contamination from wild birds might have introduced LPAIV H7N7 into the red-crowned crane at the zoo. Therefore, our results emphasize that enhanced biosecurity measures should be employed during the wild bird migration season, and that continued surveillance should be undertaken to prevent potential threats to avian species in zoos and to humans.

**Supplementary Information:**

The online version contains supplementary material available at 10.1186/s12917-020-02645-4.

## Background

Influenza A virus belongs to the *Orthomyxoviridae* family of RNA viruses, which comprise 18 hemagglutinin (HA) and 11 neuraminidase (NA) subtypes [1]. Of these, 1–16 HA subtypes and 1–9 NA subtypes have been detected in avian species [1, 2]. Wild waterfowl are natural reservoirs of AIVs, which are occasionally transmitted to domestic poultry [3]. Outbreaks of AIVs in poultry result in serious animal health and economic problems [2, 3]. In addition, some AIVs, particularly H5 and H7 subtypes, have sporadically infected mammalian hosts, resulting in potential cross-species (zoonotic) infections [4].

According to the world organization for animal health [5], AIVs can be classified as highly pathogenic and low pathogenic avian influenza virus (HPAIV and LPAIV, respectively). Some LPAIVs, mostly notably the H5 and H7 subtypes, have the potential to mutate into HPAIVs. Considering that AIVs can be transmitted from wild birds to poultry, surveillance of AIVs is required to prevent outbreaks of HPAIVs in domestic birds [3]. In South Korea, numerous low pathogenic avian influenza (LPAI) H7 viruses have been detected in wild birds [6, 7]. On rare occasions between 2009 and 2011, LPAI H7 viruses had been isolated in poultry in South Korea [8]. In March 2017, a LPAI H7N7 virus was isolated for the first time from fecal samples collected from an outdoor aviary flight cage in a zoo.

Here, we analyzed the phylogenetic and genetic relationship between the LPAI H7N7 virus isolated from the zoo and LPAI H7N7 viruses detected in wild birds within the same time period. The aim was to identify possible sources of LPAI H7N7 virus in the zoo.

## Results and discussion

We isolated a LPAI H7N7 virus from feces collected from an outdoor aviary flight cage in a zoo. DNA barcoding analysis identified the viral host as the red-crowned crane (family *Gruidae*) in bird cage in a zoo. The isolated LPAI H7N7 virus was designated A/red-crowned crane/Korea/H1026/2017 (referred to hereafter as H1026 virus). The amino acid sequence of the H1026 HA protein contained a monobasic arginine residue at the cleavage site (PELPKGR/GLF), which is known to be consistent with that in a low-pathogenicity phenotype in chickens (Table 1) [13].

**Table 1:** Molecular characteristics associated with pathogenicity of H7 isolates

Virus	Subtype	Pathotype	HA^a^				NA^b^		PB2		PB1-F2	M1	M2	NS1	Reference
			Cleavage site	RBS			294	Del	627	701	66	15	31	42	
				186	226	228									
A/mallard/Kr/H982–6/2017	H7N7	LPAI	PELPKGR	G	Q	G	N	No	E	D	N	V	S	S	[9]
**A/red-crowned crane/Kr/H1026/2017**	H7N7	LPAI	PELPKGR	G	Q	G	N	No	E	D	N	V	S	S	**This study**
A/mallard/Kr/H1029–5/2017	H7N7	LPAI	PELPKGR	G	Q	G	N	No	E	D	N	V	S	S	[9]
A/mallard/Kr/H1065–1/2017	H7N7	LPAI	PELPKGR	G	Q	G	N	No	E	D	N	V	S	S	[9]
A/mallard/Kr/H1066–5/2017	H7N7	LPAI	PELPKGR	G	Q	G	N	No	E	D	N	V	S	S	[9]
A/Netherlands/219/2003	H7N7	HPAI	PEIPKRRRR	G	Q	G	N	No	K	D	N	V	S	S	[10]
A/Italy/3/2013	H7N7	HPAI	PETPKRRERR	G	Q	G	N	No	E	D	N	V	S	S	[11]
A/Anhui/1/2013	H7N9	LPAI	PEIPKGR	V	L	G	R	YES	K	D	N	I	N	S	[12]

*HA* Hemagglutinin, *HPAI* Highly pathogenic avian influenza, *LPAI* Low pathogenic avian influenza, *NA* Neuraminidase, *PB* Polymerase basic, *PA* Polymerase acidic, *M* Matrix, *NS* Nonstructural, *RBS* RNA binding site

^a^H3 and ^b^N1 numbering was used

**Fig. 1:**
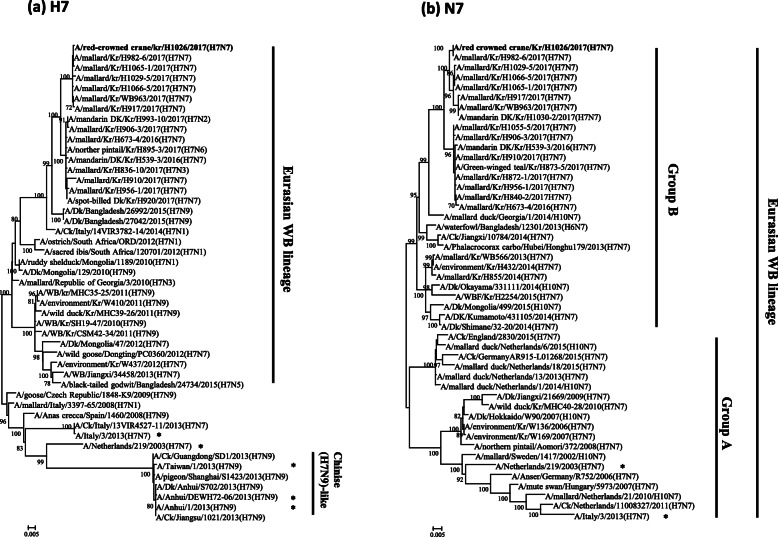
The maximum likelihood phylogenetic trees for the H7 (**a**) and N7 (**b**) gene segments of AIVs isolated from wild bird feces during national active surveillance between 2016 and 2017. The virus isolated from red-crowned crane feces is indicated in bold font. Cases of human infection with H7 virus are marked with asterisks. The scale bars represent the number of substitutions per nucleotide. Branch labels record the stability of the branches over 1000 bootstrap replicates. Only bootstrap values > 70% are shown in each tree

**Fig. 2:**
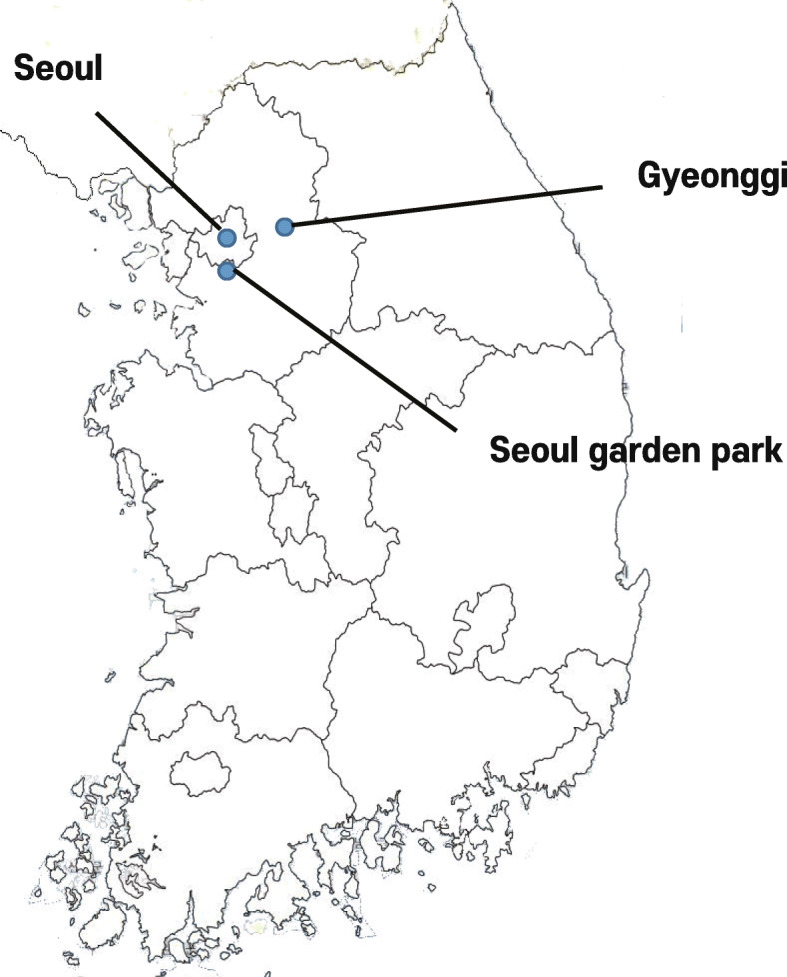
The sampling sites of H7N7 viruses in March 2017. Blue dots indicate the regions in which the viruses were isolated. Details of sample collections and processing are indicated in the section “Methods”. *The map was created by our own

Phylogenetic analysis demonstrated that the H7 gene of the H1026 virus belongs to a Eurasian wild bird lineage and distinguished from the H7N7 HPAIVs that caused human infections in Europe and Chinese H7N9-like viruses (Fig. 1a). Furthermore, the HA gene of the H1026 virus is closely related to LPAI H7N7 viruses isolated from wild bird habitats in South Korea in the winter March 2017 season (~ 99.71–99.94% homology) (Table 2). The N7 genes from Eurasian wild bird lineages were divided into groups A and B (Fig. 1b). Group A comprised viruses detected in Eurasia from 2006 to 2016. Group B comprised N7 viruses recently identified in Asian countries between 2013 and 2017 (including the H1026 virus). The NA gene of the H1026 virus was highly similar to that of four LPAI H7N7 viruses (~ 99.23–99.72% at the nucleotide level) (Table 2). Gene constellation analysis revealed that the genotype of the H1026 virus was identical to the genotype of four LPAI H7N7 viruses isolated from feces collected from wild bird habitats in nearby regions (Seoul and Gyeonggi) in March 2017 (Table 2). Seven of the eight gene fragments of the H1026 virus (the PA gene was the exception) showed high nucleotide sequence similarity (~ 99.12–100%) with that of four LPAI H7N7 viruses (Table 2). However, PA gene of the H1026 virus shared a nucleotide sequence homology (~ 97.50–98.00%) to that of four LPAI H7N7 viruses (Supplementary Table 1). Considering that nucleotide similarity and branch length for PA gene of the H1026 virus with that of four LPAI H7N7 viruses, it is likely that the unsampled ancestor, sharing > 98% high similarity in PA gene, could exist in wild bird populations (Supplementary Fig. 1c). The wild bird habitats from which the four LPAI H7N7 viruses isolated were located in the western part of South Korea (Fig. 2). It has been previously reported that the western part of South Korea, including Seoul and Gyeonggi, is overwintering or stopover site for migratory birds [14, 15]. In addition, H7 subtypes were predominantly isolated from wild bird habitats in the 2016/2017 winter season, which is unusual [9]. Considering that under the circumstances for open cage or without top roof cover in outdoor aviary flight cage in zoo, as well as the finding of high similarity between the genes of the isolated viruses and between the collection dates of the viruses, it is likely that fecal contamination from wild birds might have introduced the LPAI H7N7 virus into red-crowned cranes in the zoo.

**Table 2:** List of H7N7 low pathogenic avian influenza viruses isolated from Seoul and Gyeonggi in March 2017

Virus name	Subtype	Pathotype	Collection date	Region	Homology (%)	GISAID Isolate ID	Reference
A/mallard/Kr/H982–6/2017	H7N7	LPAI	2017-03-06	Seoul	97.59–100	EPI_ISL_309201	[9]
**A/red-crowned crane/Kr/H1026/2017**	**H7N7**	**LPAI**	**2017-03-09**	**Seoul**	100	**EPI_ISL_398128**	**This study**
A/mallard/Kr/H1029–5/2017	H7N7	LPAI	2017-03-13	GG	97.81–100	EPI_ISL_309202	[9]
A/mallard/Kr/H1065–1/2017	H7N7	LPAI	2017-03-21	Seoul	98–99.96	EPI_ISL_309203	[9]
A/mallard/Kr/H1066–5/2017	H7N7	LPAI	2017-03-20	GG	97.95–100	EPI_ISL_309204	[9]

*GISAID* Global initiative on sharing all influenza data, *GG* Gyeonggi-do, *LPAI* Low pathogenic avian influenza

HPAIVs had been detected sporadically in zoos in many countries [16–18]. In a well-known case of zoo infection, various waterfowl on an open pond at two zoological parks in Hong Kong were infected with HPAIV H5N1 in 2002 [16]. Between 2014 and 2015, clade 2.3.4.4 HPAIV H5N8 was detected in white storks in a German zoo [17], and in the winter of 2016, two Japanese zoos reported infection of black swans and snowy owls by clade 2.3.4.4 HPAIV H5N6 [18]. Of these, the highly pathogenic avian influenza (HPAI) H5N6 viruses in the Japanese zoos were isolated from avian species listed as having “least concern status” (i.e., black swans), as classified by the International Union for Conservation of Nature 3.1 (IUCN), and they were isolated from “threatened species” (i.e., snowy owl) [18, 19]. According to the IUCN’s Red List, red-crowned cranes are classified as endangered and their numbers continue to decrease [19]. Previous reports isolated HPAI H5N6 virus from debilitated or dead cranes, and LPAI H6N2 and H11N9 viruses from crane feces in wild bird habitats [20, 21]; this suggests that the crane was likely susceptible to circulating AIVs. Therefore, protecting endangered species in zoos against infection by AIVs may require vaccination, administration of antiviral drugs, and additional biosecurity measures.

During an outbreak of HPAI H7N7 virus that began in poultry farms in the Netherlands in 2003, the virus was transmitted from birds to humans with a fatal outcome [10]. An outbreak of HPAI H7N7 virus was reported in poultry in Italy in 2013, and the virus was subsequently detected in poultry workers [11]. Since 2013, human infections by Chinese H7N9-like strains have become a concern as some cases have led to severe illness and death [12]. To investigate the pathogenic potential of the H1026 virus in mammals, we examined its molecular characteristics by comparing the deduced amino acid sequences at particular sites known to be related to virulence, host tropism, and drug resistance. The molecular characteristics of the H1026 virus are different from those of the Chinese H7N9 virus (Table 1). Well-known key molecular markers associated with mammalian adaptation and pathogenicity, such as 186 V and 226 L in HA, deletion of the stalk region in NA, and E627K and D701N substitutions in PB2, were not detected in the LPAI H7N7 viruses [12]. However, a single amino acid substitution (P42S) was detected in the NS1 protein; this substitution is associated with increased pathogenicity in mice [22], which was also founded in other H7 isolates reported previously [9, 23]. By contrast, the molecular determinants of the H1026 virus (except for the cleavage motif in the HA protein and the substitution at position 627 in PB2), were similar to those of HPAI H7N7 viruses associated with human infections in Europe (Table 1). The major symptoms of HPAI H7N7 virus infection in humans, with the exception of one case of death are mild conjunctivitis and an influenza-like illness [11, 24]. Thus, continued surveillance is required to monitor the spread of zoonotic AIVs (especially the H7 subtype) that may pose a potential threat to animal and human health.

## Conclusion

In this study, LPAI H7N7 virus was isolated form feces samples collected from an outdoor aviary flight cage in a zoo in South Korea. It is likely that fecal contamination from wild birds was introduced to susceptible birds in the zoo in winter season. Therefore, we suggest that enhanced biosecurity measures should be put in place. AI surveillance and prevention strategies during the winter migration season should help to reduce potential threats to avian species in zoos and to humans.

## Methods

### Virus isolation and DNA barcoding system

South Korea conducts annual national surveillance programs to detect AI in domestic poultry, live bird markets, and wild birds [9]. In March 2017, fecal samples were collected from an outdoor aviary flight cage in Seoul Garden Park and submitted to the Seoul Research Institute of Public Health and Environment. One H7-positive sample was detected by real-time reverse transcription polymerase chain reaction (RT-PCR.) This sample was transferred to the Animal and Plant Quarantine Agency (APQA). The sample was suspended in phosphate-buffered saline containing gentamicin and inoculated into the allantoic cavity of 9–11-day-old specific-pathogen-free embryonated eggs. After a 96 h incubation at 37 °C, the eggs were chilled, and the allantoic fluid was harvested and tested for hemagglutination activity using chicken erythrocytes. Viral RNA was extracted from allantoic fluid using a Patho Gene-Spin Viral DNA/RNA extraction kit (Intron Biotechnology, Seongnam, South Korea). A barcoding system utilizing mitochondrial DNA from fecal specimens was employed to determine the host species of the AIV, as previously described [25].

### Sequencing

Viral RNA segments were converted to complimentary DNA (cDNA) by RT-PCR using an Omniscript reverse transcription kit (QIAGEN, Germantown, MD, USA). Next, cDNA segments were amplified by polymerase chain reaction using universal and gene-specific primers plus Ex Taq polymerase (TAKARA, Kusatsu, Japan). Segments were sequenced using an ABI 3730xl DNA analyzer (Applied Biosystems, Foster City, CA, USA) [26] and were assigned to the Global Initiative on Sharing All Influenza Data (GISAID) and National Center for Biotechnology Information (NCBI), respectively.

### Phylogenetic analyses

Gene-segment-specific phylogenetic trees were built using the maximum-likelihood method within MEGA (version 6.0) software [27]. The Hasegawa-Kishino-Yano model with gamma distribution (HKY + G) were determined for the Bayesian Information Criterion (BIC) and the maximum-likelihood value, and selected as the best-fitted nucleotide substitution models for H7 and N7 gene segments [28]. Reference sequences from other countries used in the phylogenetic analysis were downloaded from the National Center for Biotechnology Information Influenza Virus Resource and GISAID.

### Gene constellation analysis

Genotypes were defined according to the gene-segment-specific phylogenetic trees. A cluster was regarded as monophyletic only when it had a bootstrap support value > 70 and a nucleotide sequence identity > 97%. Each genotype comprised a combined cluster assignment of eight individual gene segments.

## Supplementary Information

**Additional file 1: Supplementary Figure 1.** The maximum likelihood phylogenetic trees for the PB2 (a), PB1 (b), PA (c), NP (d), M (e) and NS (f) gene segments of the AIVs isolated from wild bird feces in national active surveillance between 2016 and 2017. The virus isolated from red crowned crane feces was indicated in red. The scale bars represent the number of substitutions per nucleotide. Branch labels record the stability of the branches over 1000 bootstrap replicates. Only bootstrap values > 70% are shown in each tree.

**Additional file 2: Supplementary Table 1.** Nucleotide similarity for each segment between low pathogenic avian influenza H7N7 isolated from a zoo and wild bird habitats.

## Data Availability

In this study, A/red-crowned crane/Korea/H1026/2017 (H7N7) strain was isolated from a zoo in South Korea. The nucleotide sequences of the characterized isolate in this study corresponding to PB2, PB1, PA, HA, NP, NA, M and NS genes were submitted to the GISAID EpiFlu database (GISAID Isolate ID: EPI_ISL_398128) and NCBI Genbank database (Accession numbers: MW116743–MW116750), respectively. The GISAID EpiFlu database and NCBI Genbank database provides public open-access to the most complete collection of genetic sequence data of influenza viruses.

## Abbreviations

AI: Avian influenza
AIV: Avian influenza virus
APQA: Animal and Plant Quarantine Agency
BIC: Bayesian Information Criterion
cDNA: Complimentary DNA
GISAID: Global Initiative on Sharing All Influenza Data
HA: Hemagglutinin
HKY + G: Hasegawa-Kishino-Yano model with gamma distribution
HPAI: Highly pathogenic avian influenza
HPAIV: Highly pathogenic avian influenza virus
IUCN: International Union for Conservation of Nature
LPAI: Low pathogenic avian influenza
LPAIV: Low pathogenic avian influenza virus
MAFRA: Ministry of Agriculture, Food and Rural Affairs
RT-PCR: Reverse transcription polymerase chain reaction
