# Supplementary material for: Isolation and characterization of low pathogenic H7N7 avian influenza virus from a red-crowned crane in a zoo in South Korea
Source: BMC Vet Res. 2020 Nov 10;16:432. doi: 10.1186/s12917-020-02645-4 (PMC7653808; doi:10.1186/s12917-020-02645-4)
Supplement: Supplementary file 1 — Additional file 1: Supplementary Figure 1. The maximum likelihood phylogenetic trees for the PB2 (a), PB1 (b), PA (c), NP (d), M (e) and NS (f) gene segments of the AIVs isolated from wild bird feces in national active surveillance between 2016 and 2017. The virus isolated from red crowned crane feces was indicated in red. The scale bars represent the number of substitutions per nucleotide. Branch labels record the stability of the branches over 1000 bootstrap replicates. Only bootstrap values > 70% are shown in each tree. [file 12917_2020_2645_MOESM1_ESM.pptx]

## Slide 1
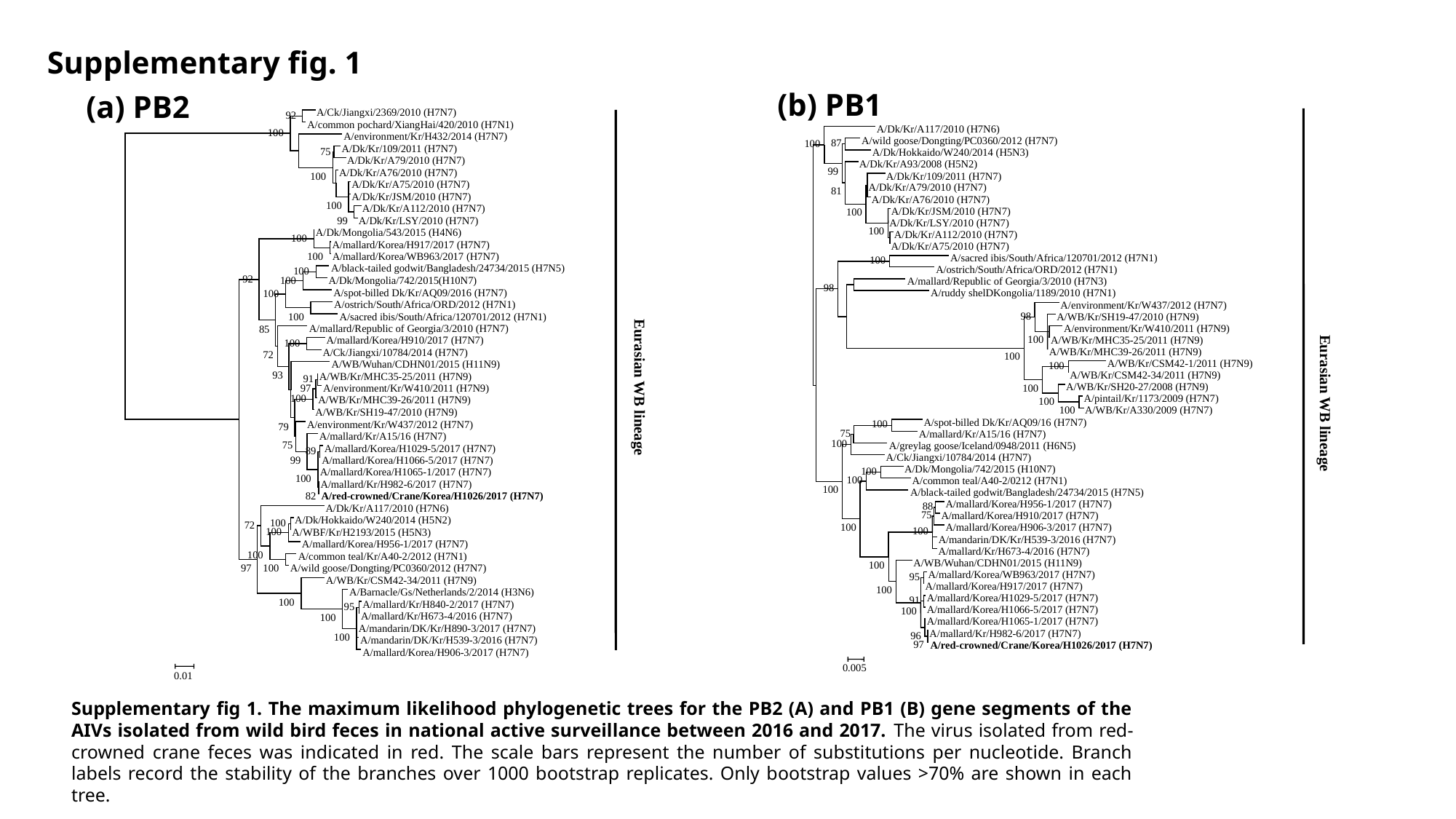

Supplementary fig. 1
(b) PB1
(a) PB2
A/Ck/Jiangxi/2369/2010 (H7N7)
92
A/common pochard/XiangHai/420/2010 (H7N1)
100
A/environment/Kr/H432/2014 (H7N7)
A/Dk/Kr/109/2011 (H7N7)
75
A/Dk/Kr/A79/2010 (H7N7)
A/Dk/Kr/A76/2010 (H7N7)
100
A/Dk/Kr/A75/2010 (H7N7)
A/Dk/Kr/JSM/2010 (H7N7)
100
A/Dk/Kr/A112/2010 (H7N7)
99
A/Dk/Kr/LSY/2010 (H7N7)
A/Dk/Mongolia/543/2015 (H4N6)
100
A/mallard/Korea/H917/2017 (H7N7)
A/mallard/Korea/WB963/2017 (H7N7)
100
A/black-tailed godwit/Bangladesh/24734/2015 (H7N5)
100
92
100
A/Dk/Mongolia/742/2015(H10N7)
A/spot-billed Dk/Kr/AQ09/2016 (H7N7)
100
A/ostrich/South/Africa/ORD/2012 (H7N1)
100
A/sacred ibis/South/Africa/120701/2012 (H7N1)
A/mallard/Republic of Georgia/3/2010 (H7N7)
85
A/mallard/Korea/H910/2017 (H7N7)
100
A/Ck/Jiangxi/10784/2014 (H7N7)
72
A/WB/Wuhan/CDHN01/2015 (H11N9)
93
A/WB/Kr/MHC35-25/2011 (H7N9)
91
97
A/environment/Kr/W410/2011 (H7N9)
100
A/WB/Kr/MHC39-26/2011 (H7N9)
A/WB/Kr/SH19-47/2010 (H7N9)
A/environment/Kr/W437/2012 (H7N7)
79
A/mallard/Kr/A15/16 (H7N7)
75
A/mallard/Korea/H1029-5/2017 (H7N7)
89
99
A/mallard/Korea/H1066-5/2017 (H7N7)
A/mallard/Korea/H1065-1/2017 (H7N7)
100
A/mallard/Kr/H982-6/2017 (H7N7)
82
A/red-crowned/Crane/Korea/H1026/2017 (H7N7)
A/Dk/Kr/A117/2010 (H7N6)
A/Dk/Hokkaido/W240/2014 (H5N2)
100
72
100
A/WBF/Kr/H2193/2015 (H5N3)
A/mallard/Korea/H956-1/2017 (H7N7)
100
A/common teal/Kr/A40-2/2012 (H7N1)
97
100
A/wild goose/Dongting/PC0360/2012 (H7N7)
A/WB/Kr/CSM42-34/2011 (H7N9)
A/Barnacle/Gs/Netherlands/2/2014 (H3N6)
100
A/mallard/Kr/H840-2/2017 (H7N7)
95
A/mallard/Kr/H673-4/2016 (H7N7)
100
A/mandarin/DK/Kr/H890-3/2017 (H7N7)
100
A/mandarin/DK/Kr/H539-3/2016 (H7N7)
A/mallard/Korea/H906-3/2017 (H7N7)
0.01
A/Dk/Kr/A117/2010 (H7N6)
A/wild goose/Dongting/PC0360/2012 (H7N7)
87
100
A/Dk/Hokkaido/W240/2014 (H5N3)
A/Dk/Kr/A93/2008 (H5N2)
99
A/Dk/Kr/109/2011 (H7N7)
A/Dk/Kr/A79/2010 (H7N7)
81
A/Dk/Kr/A76/2010 (H7N7)
A/Dk/Kr/JSM/2010 (H7N7)
100
A/Dk/Kr/LSY/2010 (H7N7)
100
A/Dk/Kr/A112/2010 (H7N7)
A/Dk/Kr/A75/2010 (H7N7)
A/sacred ibis/South/Africa/120701/2012 (H7N1)
100
A/ostrich/South/Africa/ORD/2012 (H7N1)
A/mallard/Republic of Georgia/3/2010 (H7N3)
98
A/ruddy shelDKongolia/1189/2010 (H7N1)
A/environment/Kr/W437/2012 (H7N7)
98
A/WB/Kr/SH19-47/2010 (H7N9)
A/environment/Kr/W410/2011 (H7N9)
100
A/WB/Kr/MHC35-25/2011 (H7N9)
A/WB/Kr/MHC39-26/2011 (H7N9)
100
A/WB/Kr/CSM42-1/2011 (H7N9)
100
A/WB/Kr/CSM42-34/2011 (H7N9)
A/WB/Kr/SH20-27/2008 (H7N9)
100
A/pintail/Kr/1173/2009 (H7N7)
100
100
A/WB/Kr/A330/2009 (H7N7)
A/spot-billed Dk/Kr/AQ09/16 (H7N7)
100
75
A/mallard/Kr/A15/16 (H7N7)
100
A/greylag goose/Iceland/0948/2011 (H6N5)
A/Ck/Jiangxi/10784/2014 (H7N7)
A/Dk/Mongolia/742/2015 (H10N7)
100
100
A/common teal/A40-2/0212 (H7N1)
100
A/black-tailed godwit/Bangladesh/24734/2015 (H7N5)
A/mallard/Korea/H956-1/2017 (H7N7)
88
75
A/mallard/Korea/H910/2017 (H7N7)
100
A/mallard/Korea/H906-3/2017 (H7N7)
100
A/mandarin/DK/Kr/H539-3/2016 (H7N7)
A/mallard/Kr/H673-4/2016 (H7N7)
A/WB/Wuhan/CDHN01/2015 (H11N9)
100
A/mallard/Korea/WB963/2017 (H7N7)
95
A/mallard/Korea/H917/2017 (H7N7)
100
A/mallard/Korea/H1029-5/2017 (H7N7)
91
A/mallard/Korea/H1066-5/2017 (H7N7)
100
A/mallard/Korea/H1065-1/2017 (H7N7)
A/mallard/Kr/H982-6/2017 (H7N7)
96
97
A/red-crowned/Crane/Korea/H1026/2017 (H7N7)
0.005
Eurasian WB lineage
Eurasian WB lineage
Supplementary fig 1. The maximum likelihood phylogenetic trees for the PB2 (A) and PB1 (B) gene segments of the AIVs isolated from wild bird feces in national active surveillance between 2016 and 2017. The virus isolated from red-crowned crane feces was indicated in red. The scale bars represent the number of substitutions per nucleotide. Branch labels record the stability of the branches over 1000 bootstrap replicates. Only bootstrap values >70% are shown in each tree.

## Slide 2
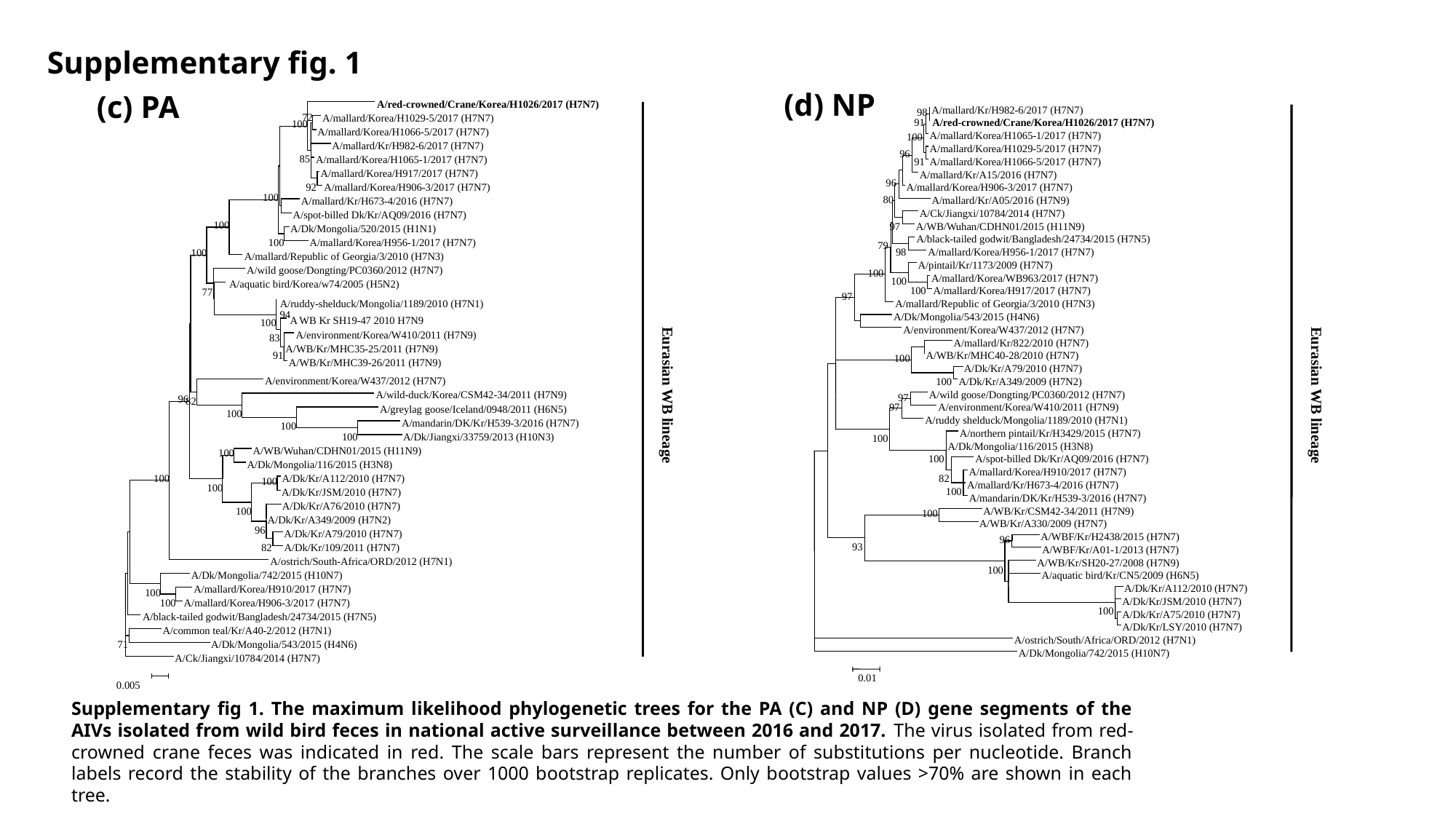

Supplementary fig. 1
(d) NP
(c) PA
A/mallard/Kr/H982-6/2017 (H7N7)
98
91
A/red-crowned/Crane/Korea/H1026/2017 (H7N7)
A/mallard/Korea/H1065-1/2017 (H7N7)
100
A/mallard/Korea/H1029-5/2017 (H7N7)
96
91
A/mallard/Korea/H1066-5/2017 (H7N7)
A/mallard/Kr/A15/2016 (H7N7)
96
A/mallard/Korea/H906-3/2017 (H7N7)
80
A/mallard/Kr/A05/2016 (H7N9)
A/Ck/Jiangxi/10784/2014 (H7N7)
97
A/WB/Wuhan/CDHN01/2015 (H11N9)
A/black-tailed godwit/Bangladesh/24734/2015 (H7N5)
79
98
A/mallard/Korea/H956-1/2017 (H7N7)
A/pintail/Kr/1173/2009 (H7N7)
100
A/mallard/Korea/WB963/2017 (H7N7)
100
100
A/mallard/Korea/H917/2017 (H7N7)
97
A/mallard/Republic of Georgia/3/2010 (H7N3)
A/Dk/Mongolia/543/2015 (H4N6)
A/environment/Korea/W437/2012 (H7N7)
A/mallard/Kr/822/2010 (H7N7)
A/WB/Kr/MHC40-28/2010 (H7N7)
100
A/Dk/Kr/A79/2010 (H7N7)
100
A/Dk/Kr/A349/2009 (H7N2)
A/wild goose/Dongting/PC0360/2012 (H7N7)
97
97
A/environment/Korea/W410/2011 (H7N9)
A/ruddy shelduck/Mongolia/1189/2010 (H7N1)
A/northern pintail/Kr/H3429/2015 (H7N7)
100
A/Dk/Mongolia/116/2015 (H3N8)
100
A/spot-billed Dk/Kr/AQ09/2016 (H7N7)
A/mallard/Korea/H910/2017 (H7N7)
82
A/mallard/Kr/H673-4/2016 (H7N7)
100
A/mandarin/DK/Kr/H539-3/2016 (H7N7)
A/WB/Kr/CSM42-34/2011 (H7N9)
100
A/WB/Kr/A330/2009 (H7N7)
A/WBF/Kr/H2438/2015 (H7N7)
96
93
A/WBF/Kr/A01-1/2013 (H7N7)
A/WB/Kr/SH20-27/2008 (H7N9)
100
A/aquatic bird/Kr/CN5/2009 (H6N5)
A/Dk/Kr/A112/2010 (H7N7)
A/Dk/Kr/JSM/2010 (H7N7)
100
A/Dk/Kr/A75/2010 (H7N7)
A/Dk/Kr/LSY/2010 (H7N7)
A/ostrich/South/Africa/ORD/2012 (H7N1)
A/Dk/Mongolia/742/2015 (H10N7)
0.01
A/red-crowned/Crane/Korea/H1026/2017 (H7N7)
72
A/mallard/Korea/H1029-5/2017 (H7N7)
100
A/mallard/Korea/H1066-5/2017 (H7N7)
A/mallard/Kr/H982-6/2017 (H7N7)
85
A/mallard/Korea/H1065-1/2017 (H7N7)
A/mallard/Korea/H917/2017 (H7N7)
92
A/mallard/Korea/H906-3/2017 (H7N7)
100
A/mallard/Kr/H673-4/2016 (H7N7)
A/spot-billed Dk/Kr/AQ09/2016 (H7N7)
100
A/Dk/Mongolia/520/2015 (H1N1)
100
A/mallard/Korea/H956-1/2017 (H7N7)
100
A/mallard/Republic of Georgia/3/2010 (H7N3)
A/wild goose/Dongting/PC0360/2012 (H7N7)
 A/aquatic bird/Korea/w74/2005 (H5N2)
77
A/ruddy-shelduck/Mongolia/1189/2010 (H7N1)
94
 A WB Kr SH19-47 2010 H7N9
100
A/environment/Korea/W410/2011 (H7N9)
83
A/WB/Kr/MHC35-25/2011 (H7N9)
91
A/WB/Kr/MHC39-26/2011 (H7N9)
A/environment/Korea/W437/2012 (H7N7)
A/wild-duck/Korea/CSM42-34/2011 (H7N9)
96
82
A/greylag goose/Iceland/0948/2011 (H6N5)
100
A/mandarin/DK/Kr/H539-3/2016 (H7N7)
100
100
A/Dk/Jiangxi/33759/2013 (H10N3)
A/WB/Wuhan/CDHN01/2015 (H11N9)
100
A/Dk/Mongolia/116/2015 (H3N8)
100
A/Dk/Kr/A112/2010 (H7N7)
100
100
A/Dk/Kr/JSM/2010 (H7N7)
A/Dk/Kr/A76/2010 (H7N7)
100
A/Dk/Kr/A349/2009 (H7N2)
96
A/Dk/Kr/A79/2010 (H7N7)
82
A/Dk/Kr/109/2011 (H7N7)
A/ostrich/South-Africa/ORD/2012 (H7N1)
A/Dk/Mongolia/742/2015 (H10N7)
A/mallard/Korea/H910/2017 (H7N7)
100
100
A/mallard/Korea/H906-3/2017 (H7N7)
A/black-tailed godwit/Bangladesh/24734/2015 (H7N5)
A/common teal/Kr/A40-2/2012 (H7N1)
71
A/Dk/Mongolia/543/2015 (H4N6)
A/Ck/Jiangxi/10784/2014 (H7N7)
0.005
Eurasian WB lineage
Eurasian WB lineage
Supplementary fig 1. The maximum likelihood phylogenetic trees for the PA (C) and NP (D) gene segments of the AIVs isolated from wild bird feces in national active surveillance between 2016 and 2017. The virus isolated from red-crowned crane feces was indicated in red. The scale bars represent the number of substitutions per nucleotide. Branch labels record the stability of the branches over 1000 bootstrap replicates. Only bootstrap values >70% are shown in each tree.

## Slide 3
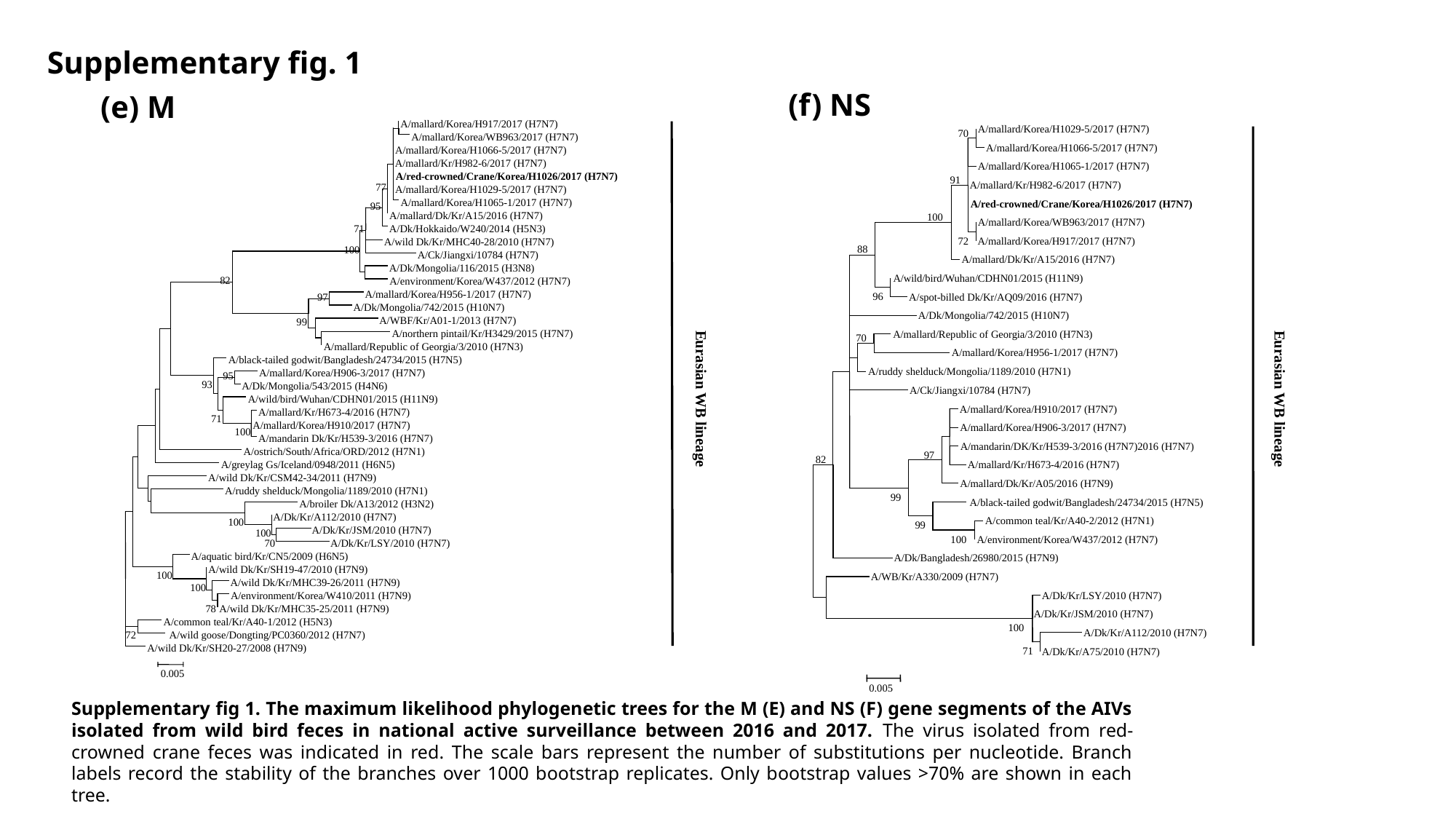

Supplementary fig. 1
(f) NS
(e) M
A/mallard/Korea/H917/2017 (H7N7)
A/mallard/Korea/WB963/2017 (H7N7)
A/mallard/Korea/H1066-5/2017 (H7N7)
A/mallard/Kr/H982-6/2017 (H7N7)
A/red-crowned/Crane/Korea/H1026/2017 (H7N7)
77
A/mallard/Korea/H1029-5/2017 (H7N7)
A/mallard/Korea/H1065-1/2017 (H7N7)
95
A/mallard/Dk/Kr/A15/2016 (H7N7)
71
A/Dk/Hokkaido/W240/2014 (H5N3)
A/wild Dk/Kr/MHC40-28/2010 (H7N7)
100
A/Ck/Jiangxi/10784 (H7N7)
A/Dk/Mongolia/116/2015 (H3N8)
82
A/environment/Korea/W437/2012 (H7N7)
A/mallard/Korea/H956-1/2017 (H7N7)
97
A/Dk/Mongolia/742/2015 (H10N7)
A/WBF/Kr/A01-1/2013 (H7N7)
99
A/northern pintail/Kr/H3429/2015 (H7N7)
A/mallard/Republic of Georgia/3/2010 (H7N3)
A/black-tailed godwit/Bangladesh/24734/2015 (H7N5)
A/mallard/Korea/H906-3/2017 (H7N7)
95
93
A/Dk/Mongolia/543/2015 (H4N6)
A/wild/bird/Wuhan/CDHN01/2015 (H11N9)
A/mallard/Kr/H673-4/2016 (H7N7)
71
A/mallard/Korea/H910/2017 (H7N7)
100
A/mandarin Dk/Kr/H539-3/2016 (H7N7)
A/ostrich/South/Africa/ORD/2012 (H7N1)
A/greylag Gs/Iceland/0948/2011 (H6N5)
A/wild Dk/Kr/CSM42-34/2011 (H7N9)
A/ruddy shelduck/Mongolia/1189/2010 (H7N1)
A/broiler Dk/A13/2012 (H3N2)
A/Dk/Kr/A112/2010 (H7N7)
100
A/Dk/Kr/JSM/2010 (H7N7)
100
70
A/Dk/Kr/LSY/2010 (H7N7)
A/aquatic bird/Kr/CN5/2009 (H6N5)
A/wild Dk/Kr/SH19-47/2010 (H7N9)
100
A/wild Dk/Kr/MHC39-26/2011 (H7N9)
100
A/environment/Korea/W410/2011 (H7N9)
78
A/wild Dk/Kr/MHC35-25/2011 (H7N9)
A/common teal/Kr/A40-1/2012 (H5N3)
72
A/wild goose/Dongting/PC0360/2012 (H7N7)
A/wild Dk/Kr/SH20-27/2008 (H7N9)
0.005
A/mallard/Korea/H1029-5/2017 (H7N7)
70
A/mallard/Korea/H1066-5/2017 (H7N7)
A/mallard/Korea/H1065-1/2017 (H7N7)
91
A/mallard/Kr/H982-6/2017 (H7N7)
A/red-crowned/Crane/Korea/H1026/2017 (H7N7)
100
A/mallard/Korea/WB963/2017 (H7N7)
72
A/mallard/Korea/H917/2017 (H7N7)
88
A/mallard/Dk/Kr/A15/2016 (H7N7)
A/wild/bird/Wuhan/CDHN01/2015 (H11N9)
96
A/spot-billed Dk/Kr/AQ09/2016 (H7N7)
A/Dk/Mongolia/742/2015 (H10N7)
A/mallard/Republic of Georgia/3/2010 (H7N3)
70
A/mallard/Korea/H956-1/2017 (H7N7)
A/ruddy shelduck/Mongolia/1189/2010 (H7N1)
A/Ck/Jiangxi/10784 (H7N7)
A/mallard/Korea/H910/2017 (H7N7)
A/mallard/Korea/H906-3/2017 (H7N7)
A/mandarin/DK/Kr/H539-3/2016 (H7N7)2016 (H7N7)
97
82
A/mallard/Kr/H673-4/2016 (H7N7)
A/mallard/Dk/Kr/A05/2016 (H7N9)
99
A/black-tailed godwit/Bangladesh/24734/2015 (H7N5)
A/common teal/Kr/A40-2/2012 (H7N1)
99
100
A/environment/Korea/W437/2012 (H7N7)
A/Dk/Bangladesh/26980/2015 (H7N9)
A/WB/Kr/A330/2009 (H7N7)
A/Dk/Kr/LSY/2010 (H7N7)
A/Dk/Kr/JSM/2010 (H7N7)
100
A/Dk/Kr/A112/2010 (H7N7)
71
A/Dk/Kr/A75/2010 (H7N7)
0.005
Eurasian WB lineage
Eurasian WB lineage
Supplementary fig 1. The maximum likelihood phylogenetic trees for the M (E) and NS (F) gene segments of the AIVs isolated from wild bird feces in national active surveillance between 2016 and 2017. The virus isolated from red-crowned crane feces was indicated in red. The scale bars represent the number of substitutions per nucleotide. Branch labels record the stability of the branches over 1000 bootstrap replicates. Only bootstrap values >70% are shown in each tree.
