# Supplementary material for: Isolation and characterization of low pathogenic H7N7 avian influenza virus from a red-crowned crane in a zoo in South Korea
Source: BMC Vet Res. 2020 Nov 10;16:432. doi: 10.1186/s12917-020-02645-4 (PMC7653808; doi:10.1186/s12917-020-02645-4)
Supplement: Supplementary file 2 — Additional file 2: Supplementary Table 1. Nucleotide similarity for each segment between low pathogenic avian influenza H7N7 isolated from a zoo and wild bird habitats. [file 12917_2020_2645_MOESM2_ESM.docx]

**Supplementary Table 1.** Nucleotide similarity for each segment between low pathogenic avian influenza H7N7 isolated from a zoo and wild bird habitats.

| Virus name | Nucleotide similarity (%) | | | | | | | | Reference |
| --- | --- | --- | --- | --- | --- | --- | --- | --- | --- |
|  | PB2 | PB1 | PA | HA | NP | NA | M | NS | [12] |
| A/mallard/Kr/H982-6/2017 | 100 | 99.12 | 97.59 | 99.94 | 99.93 | 99.72 | 100 | 100 | [12] |
| A/mallard/Kr/H1029-5/2017 | 99.57 | 99.65 | 97.82 | 99.71 | 99.61 | 99.44 | 100 | 99.88 | [12] |
| A/mallard/Kr/H1065-1/2017 | 99.96 | 99.91 | 98.00 | 99.82 | 99.74 | 99.44 | 99.90 | 99.88 | [12] |
| A/mallard/Kr/H1066-5/2017 | 99.87 | 99.65 | 97.95 | 99.76 | 99.67 | 99.23 | 100 | 99.77 | [12] |

PB, polymerase basic; PA, polymerase acidic; HA, hemagglutinin; NP; nucleocapsid protein; NA, neuraminidase; M, matrix; NS, nonstructural.
